# A Simple Bioconjugate Attachment Protocol for Use in Single Molecule Force Spectroscopy Experiments Based on Mixed Self-Assembled Monolayers

**DOI:** 10.3390/ijms131013521

**Published:** 2012-10-19

**Authors:** Simon J. Attwood, Anna M. C. Simpson, Rachael Stone, SamirW. Hamaia, Debdulal Roy, RichardW. Farndale, Myriam Ouberai, Mark E. Welland

**Affiliations:** 1 Nanoscience Centre, Department of Engineering, Cambridge University, Cambridge, CB3 0FF, UK; E-Mails: simonjamesattwood@gmail.com (S.J.A.); mmo25@cam.ac.uk (M.O.); 2 Department of Biochemistry, Cambridge University, Cambridge, CB2 1QW, UK; E-Mails: anna.simpson@cantab.net (A.M.C.S.); rj271@mole.bio.cam.ac.uk (R.S.); swh23@cam.ac.uk (S.W.H.); rwf10@cam.ac.uk (R.W.F.); 3 National Physical Laboratory, Teddington, TW11 0LW, UK; E-Mail: debdulal.roy@npl.co.uk

**Keywords:** atomic force microscopy, single molecule force spectroscopy, biotin-avidin interaction, mixed self-assembled monolayers

## Abstract

Single molecule force spectroscopy is a technique that can be used to probe the interaction force between individual biomolecular species. We focus our attention on the tip and sample coupling chemistry, which is crucial to these experiments. We utilised a novel approach of mixed self-assembled monolayers of alkanethiols in conjunction with a heterobifunctional crosslinker. The effectiveness of the protocol is demonstrated by probing the biotin-avidin interaction. We measured unbinding forces comparable to previously reported values measured at similar loading rates. Specificity tests also demonstrated a significant decrease in recognition after blocking with free avidin.

## 1. Introduction

Atomic Force Microscopy (AFM) [1] is a powerful nanoscale technique that offers piconewton sensitivity, angstrom scale resolution, and the ability to operate within a variety of environments, including those which are of physiological relevance. It is therefore well suited for the measurement of the interaction forces between individual biological molecules. By chemically modifying AFM tips and substrates, Florin *et al*. [2] first demonstrated the technique of Single Molecule Force Spectroscopy (SMFS) by measuring the biotin-avidin interaction. Later it was shown that by varying the loading rate, it is possible to probe the various transition states and kinetic off-rates that characterise the energy landscape in what is often referred to as Dynamic Force Spectroscopy [3,4] (DFS). By utilising the AFM in this manner, valuable insights into the structure and dynamics of the interacting species, which are otherwise obscured in ensemble experiments, may be obtained. So far, several different biological interactions have been studied, including biotin-avidin [5–12], biotin-streptavidin [8,13–19], antibody-antigen [20], lectin-carbohydrate [21], integrins [22], Ran-Importin *β*_1_ [23], VE-cadherins [24], selectins [25] and the adhesion force between complementary strands of DNA [18].

The interaction between biotin and avidin has attracted much attention in the field of force spectroscopy due to its highest known affinity in nature [26] (*K_a_* ≈ 10^15^M^−1^ ) and because of the wide availability of thermodynamic and structural data. There have been several articles reporting the biotin-avidin interaction, but they disagree with the kinetic off rates (*k*_off_), energy barrier widths (*x_β_*) and affinities determined from DFS experiments. For example the *x_β_* values recorded in the range ≈ 10^3^–10^4^ pN s^−1^ appear to differ approximately by an order of magnitude between the work by Piramowicz [7] and the work by De Paris [5], and the *k*_off_ values differ by approximately five orders of magnitude when comparing the works of Yuan [6] and Merkel [4] over similar loading rates.

Identifying the causes of such discrepancies is a complex problem. Inherently the unbinding force between a ligand-receptor pair modelled by a single energy barrier is not single valued but exhibits a force distribution of rupture forces with a single peak. This is due to both the stochastic nature of the unbinding event [27,28] and the heterogeneity of possible reaction pathways [29] related to the structural microheterogeneity of the complex. In addition, experimental errors deriving from many different sources broaden the distribution. If experimental errors are large, it may often be difficult to discern the fundamental unitary unbinding peak [30]. It has also been suggested that multiple simultaneous unbinding of ligand-receptor pairs may lead to artefacts in force spectra [31]. Additionally, non-specific adhesion forces may substantially distort the force spectra.

Differences in experiments may derive from the choice of AFM equipment (commercial [23], custom built [6] and hybrid setups [5]), substrates (primarily agarose beads [2,6] versus flat substrates; silicon [32], glass [33], gold [5,34]), cantilevers, calibration techniques [35–38] and the varied methods used to analyse the large amounts of data [39,40]. Most significantly however, the surface attachment protocols for coupling the biomolecules of interest to tips and substrates vary greatly between studies [2,5,10,11,34,41] (for a detailed review see [42]). The use of covalent bonds versus physisorption mediated attachment, type of passivation of surfaces employed to minimise non-specific interactions and the use of poly(ethylene glycol) (PEG) linkers [43] which improve ligand-receptor mobility, are all extremely important in these studies.

Recently Yadavalli *et al*. [44] developed a protocol using mixed self-assembled monolayers of alkanethiols on ultraflat gold surfaces. The use of ultraflat surfaces or template-stripped gold (TSG) surfaces, which may be prepared moments before use to yield flat clean substrates, has the advantage that undesirable tip interactions due to surface asperities are minimised. Also for the purposes of imaging, molecules of only a few nanometres in dimension may be distinguishable. Yadavalli covalently attached single, isolated protein molecules via lysine residues on top of a “sea” of inert PEG units to the gold substrates. This method proved very successful at minimising non-specific interactions, the density of attachment was well controlled, and they also demonstrated the stretching of rabbit myosin S2. The protocol has only recently been used for SMFS measurements between ligand-receptor pairs [45].

We expand on the work by Yadavalli using a three stage protocol, which couples biomolecules via flexible PEG crosslinkers to cysteine residues. The use of flexible tethers as opposed to rigid alkyl chains permits extra mobility of interacting groups. Also since cysteines are often less numerous in proteins than lysine residues, the possibility of site-directed coupling may be permitted providing the protein features an appropriately accessible cysteine. By simply pre-treating proteins with Traut’s Reagent (2-iminothiolane), which reacts with primary amines to introduce sulfhydryl groups [46], almost any protein is amenable to attachment with this protocol. By modifying both gold coated tips and TSG substrates in this manner we demonstrate applicability with the model ligand-receptor pair, biotin-avidin.

The use of Traut’s reagent is advantageous over *N*-succinimidyl-*S*-acetylthioacetate (SATA), succinimidyl acetylthioproionate (SATP) or *N*-Succinimidyl-3-[2-pyridyldithio]-propionate (SPDP) [42,47] since it requires no pre-derivatization or gel filtration prior to reaction with maleimide groups. Up until now it also does not appear to have been used for the purposes of SMFS experiments. Other similar approaches [42] where mixed SAMs have been employed do not utilise protein resistant groups, or target amines as opposed to sulfhydryl moieties on proteins. In other cases, although the protein linked thiol component contains PEG groups, the other thiol component contains chemical moieties at the surface that may not be so inert towards protein interactions [48]. A similar heterobifunctional crosslinker has been used for the purpose of coupling proteins to monolayers on gold surfaces [49]. However, since the orientation is reversed proteins are coupled via lysine residues, and the simpler SAM system employed is not protein-resistant.

## 2. Results and Discussion

**Figure 1:**
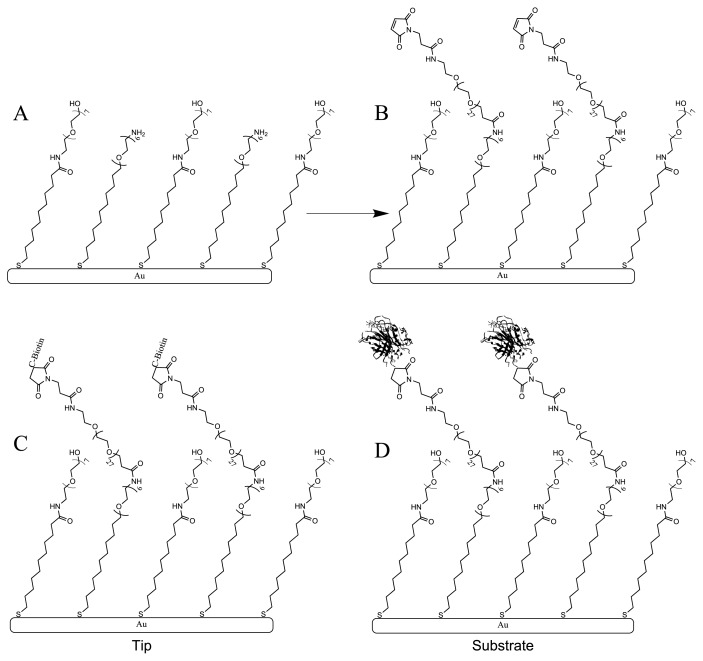
Protocol for protein attachment onto gold surfaces. **(A)** A mixed monolayer is formed from a solution containing different proportions of amine- (APA) and hydroxyl- (APH) terminating thiols; **(B)** A heterobifunctional crosslinker is then bound to the mixed SAM at available amine groups via its NHS terminus; **(C)** and **(D)** biotin and avidin respectively bound to the monolayer via sulfhydryl groups.

A schematic of the protocol devised for the covalent attachment of proteins to gold coated cantilevers and substrates is depicted in Figure 1. For the cantilevers a mixed monolayer of alkanethiols is first prepared on the gold surface (Figure 1A). Typically for force spectroscopy experiments the ratio of the amine to hydroxyl terminating thiols was 10%. By keeping the ratio of concentrations of the active to passive thiol components relatively low, a sparse coverage of protein molecules on the tip can be achieved to facilitate the measurement between individual ligand-receptor pairs. For the substrate however, a high density of protein coverage is required to maximise the chance of binding and therefore monolayers were prepared with solutions containing only the amine terminating thiol. In the second step of the protocol (Figure 1B) the *N*-hydroxysuccinimide ester (NHS) end of the heterobifunctional crosslinker is linked to the active, amine moieties of the surface bound thiol molecules. In the final step sulfhydryl groups in the proteins are linked to maleimide groups on the monolayer. Biotin was purchased with an available thiol group, but the avidin used here contains no accessible cysteine residues. It was therefore necessary to introduce sulfhydryl groups to the protein using Traut’s reagent [46]. Although such groups will likely be formed at many locations on the protein and thus orientation is difficult to control, due to the tetrameric nature of avidin this is unlikely to compromise all four binding sites. Schematics of biotin and avidin bound molecules are shown in Figure 1C,D.

One of the problems associated with solution based modification of TSG surfaces is the tendency for the adhesive between the gold surface and silicon support stub to absorb solvents, which leads to swelling and damage of the gold surface. In addition, for certain experiments it is often preferable to have only the gold surface exposed to the reactants, and although the freshly stripped gold surface is very clean, the nearby silicon and adhesive surfaces are likely to be less so. By exposing only the gold surface to the reactants all of these problems can be avoided. Wagner *et al*. [50] devised a reaction chamber for use with TSG surfaces that permits only the gold surface to come into contact with the reactants. Their system however contains several features that are not needed for the current work. As such, a new simpler device was designed. The Substrate Incubation Device (SID) was based largely on the fluid cell that is available with the PicoPlus AFM from Molecular Imaging. The only essential modification to the Agilent design is the absence of locator clips that allow the device to be attached to the AFM, and a decreased well width that was chosen to be suitable for modification of the routinely prepared TSG, which had an area of ~ 1 cm^2^. In principle therefore, if larger samples of TSG were made, substrate modification and analysis could be conducted in the same device, reducing contamination, and even allowing in-situ reaction combined with surface analysis. The SID device essentially consists of a PTFE sample holder with a circular hole through the centre, and a recessed Viton O-ring (RS electronics) that is pressed against the TSG surface. The lower half of the device consists of a rigid aluminium plate, which contains spring-loaded locking pins. One of the major advantages of this setup is that it may be assembled within moments by simply inserting the locking clips into place.

**Figure 2:**
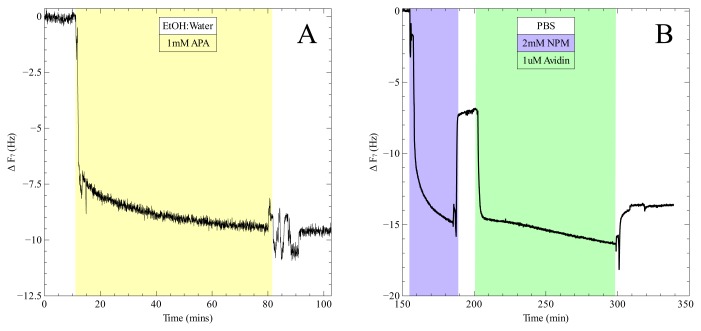
Detection of coupling steps via QCM. **(A)** Formation of APA alkanethiol monolayer on gold coated QCM chip; **(B)** Bonding of crosslinker to monolayer followed by coupling of avidin via Traut’s reagent.

The effectiveness of the new protocol was tested qualitatively by using a quartz crystal microbalance (QCM). In principle any added mass to the surface of the gold coated sensors should result in a proportional negative shift in the resonant frequency [51,52]. However, since the crosslinker and avidin are not coupled rigidly to the surface but rather protrude someway into the liquid, the sensitivity is reduced. Nevertheless we were able to detect each step of the protocol as observed by the respective shift in frequency of the seventh harmonic as shown in Figure 2. After each incubation step the QCM chamber was washed with the appropriate solvent to remove any unreacted material and allowed sufficient time to reach a stable baseline. The observed shift in frequency due to the addition of avidin (~ 7 Hz) is very similar to that of the crosslinker. Had the avidin formed a dense coverage across the surface and bound rigidly, a larger frequency shift would have been expected. However, the soft avidin molecules attached by long flexible linkers contribute less to the frequency shift. The AFM topographical data discussed next also suggests that the avidin coverage is less than 100%.

**Figure 3:**
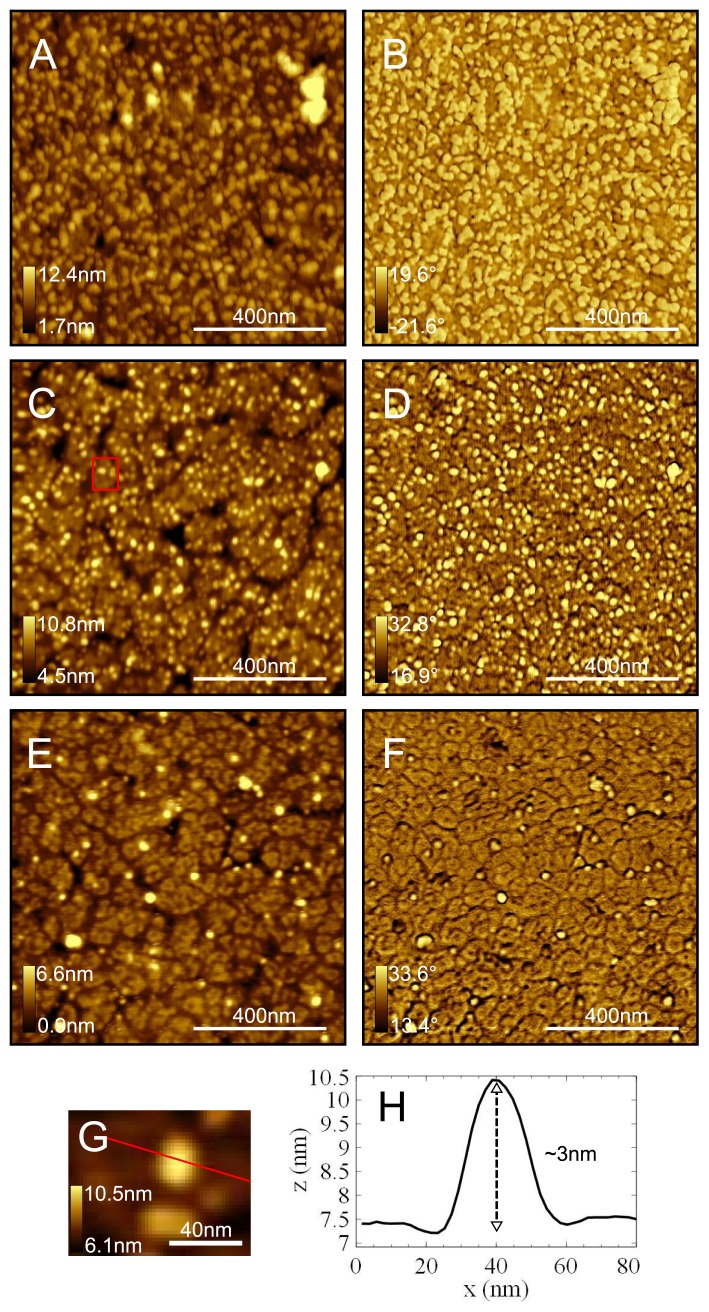
Topography (**A,C,E**) and phase (**B,D,F**) images recorded in air using AC mode. (**A,B**) Full protocol except avidin at 3 *μ*M; (**C,D**) Full protocol (avidin at 1 *μ*M); (**E,F**) Full protocol except APA replaced with APH; (**G**) Enlarged view of red box in **C**; (**H**) Height profile through red line in **G**.

In addition to the QCM measurements, AC mode AFM was used to obtain topography (Figure 3A,C,E) and phase contrast (Figure 3B,D,F) images of modified TSG surfaces. The phase images have been presented since the phase is less influenced by large topographical features but more sensitive to the influence of adhesion and changes in compliance. The presence of avidin is therefore better distinguishable from the underlying PEG layer and less influenced by the deep cracks associated with the grain boundaries of the TSG. Figure 3A,B shows a sample of TSG prepared with the full protocol except with a higher concentration of avidin (3 *μ*M), which shows a fairly high coverage but also exhibits some protein aggregation. A sample prepared using the standard protocol is shown in Figure 3C,D, which shows clearly distinguishable avidin molecules distributed across the surface and with very little aggregation as is preferable for single molecule measurements. An enlarged view of the region enclosed by the red square in Figure 3C and a height profile are shown in Figure 3G and in Figure 3H respectively. Using image thresholding analysis, we measured the average height of avidin molecules to be 2.9 ± 0.1nm (standard error, *n* = 203) as expected for avidin in air [11,53]. In Figure 3E,F the full protocol was employed except replacing the APA with APH as a control test, and as such it is expected that neither the crosslinker or the avidin would be coupled to the surface. There is a very low density of non-specifically adsorbed avidin molecules which has mostly adsorbed at the grain boundaries of the TSG.

**Figure 4:**
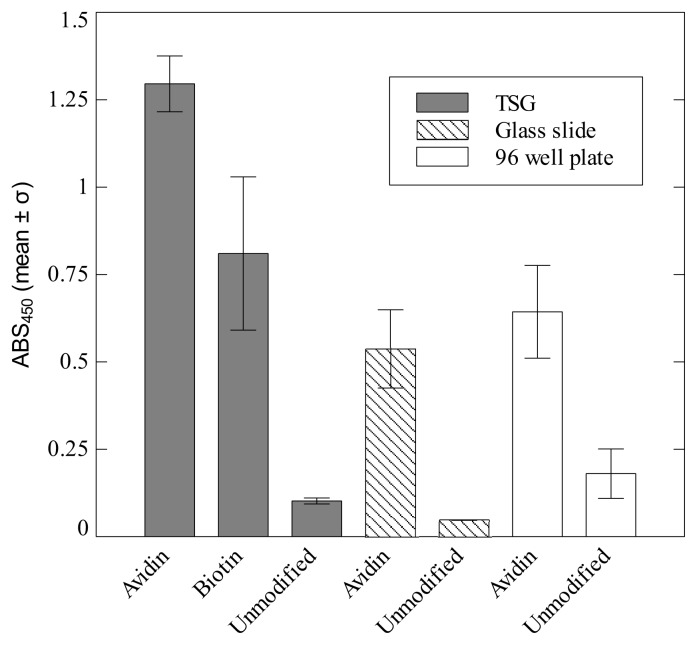
Detection via ELISA assay of the presence of either avidin or biotin bound to TSG via the monolayer (solid), avidin physisorbed to glass slides (stripes) and avidin bound to the wells of a 96-well plate (open). Avidin bound to all surfaces was readily detectable, as indicated by the significant increase in signal compared to the controls. Biotin coupled TSG was also detected. Errors are standard deviations based on 2 (TSG), 5 (glass slides) and 6 (96-well plate) replicates. Additional positive results were recorded for TSG on different days (data not shown).

To complement the QCM and AFM data, an ELISA assay protocol was developed to test for the functionality of both avidin and biotin bound via the monolayer to TSG surfaces. Avidin was detected directly using biotinylated-HRP, whereas the biotin bound layer was first incubated with avidin and then detected using biotinylated-HRP. As can be seen in Figure 4, both the avidin and biotin coupled surfaces showed a substantial increase in absorbance when compared with unmodified TSG surfaces. The biotin-bound surface showed a slightly lower response in comparison to the avidin surface, which is attributable to the indirect detection method. A blocking experiment was also conducted (data not shown) in which free biotin was added to the avidin coated surfaces so as to specifically block binding sites. An 86% reduction in absorbance was observed, confirming the specificity of the assay. Avidin was also detectable on glass slides coated with avidin using this method, although the signal is almost half as low compared with that for the monolayer-bound avidin samples. This may be simply due to the decreased area of coverage obtained when coating the glass slides using the drop-casting method as opposed to incubation within the SID. However, it is expected that the avidin bound via the monolayer will be more accessible for binding due to the flexible PEG linkers and we would therefore expect a higher signal. A standard adhesion assay conducted in an immulon-2 96-well plate again confirms that avidin is reliably detectable using this method. The combination of QCM, ELISA assay and AFM imaging data indicates first that avidin and biotin were successfully coupled to the TSG substrate via the monolayer, and secondly that they exist there in an active state, accessible for binding.

**Figure 5:**
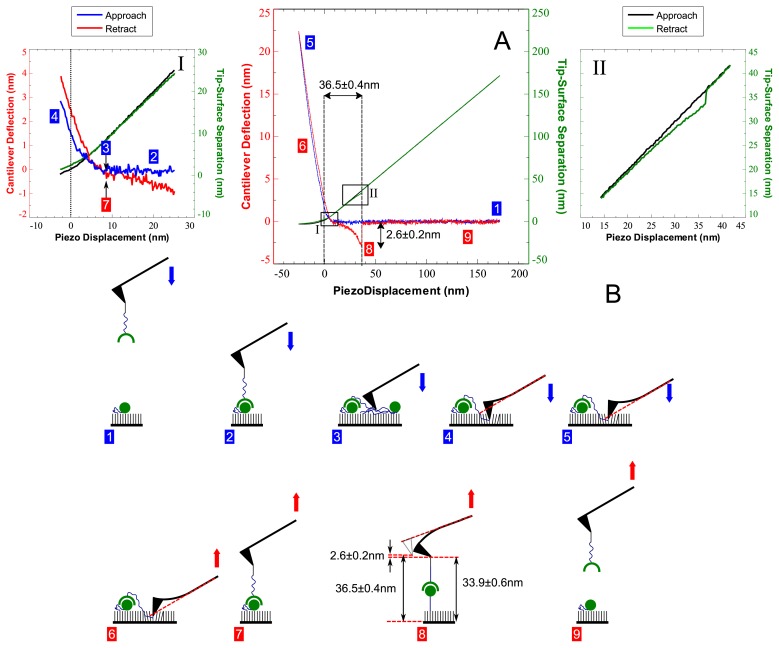
**(A)** Example biotin-avidin data set. Cantilever deflection (left vertical axis) and tip-surface separation (right vertical axis) are plotted versus the piezo displacement. Approach and retract for the deflection data are represented by the blue and red data points respectively. Approach and retract for the tip-surface separation data are represented by the black and green data points respectively. The areas enclosed by boxes I and II are enlarged in the respective sub-plots. The unbinding piezo length (*L_up_* = 36.5 ± 0.4 nm) and deflection unbinding length (*L_ud_* = 2.6 ± 0.2 nm) are also indicated. Box enclosed numbers are references for the diagrams in **(B)**. Blue and red boxed numbers again refer to approach and retract respectively. **(B)** Time series pictorial illustration of the evolution of the dynamic interactions between the tip, substrate and bound molecules.

Representative data from an approach-retract cycle recorded with tips and substrates modified using the aforementioned protocol is shown in Figure 5A together with a time-series schematic (Figure 5B). Initially the tip is far away from the substrate where it experiences no interaction forces and therefore remains undeflected as indicated by point 1. At some point in time between when the approaching tip-bound ligand is in close enough proximity to the substrate that binding may occur, and just before it leaves this position during retraction, the ligand must bind to a surface bound cognate receptor molecule (point 2). The tip initially makes contact with the material on the surface a few nanometres away from the hard substrate as indicated by point 3 in sub-plot I, after which the deflection of the cantilever starts to increase positively. Immediately preceding this point is a small non-linear deflection region indicative of inelastic compression of surface bound material. As illustrated by point 3 of the schematic diagram, this may be attributed to the compression of soft PEG linker molecules, other biomolecules or the alkane bound monolayer. After a further few nanometres the non-linear deflection curve becomes linear, indicating that the tip has reached a hard contact as implied by the negative piezo displacement (sub-plot I). This is likely to occur a short distance away from the TSG surface since the tip would otherwise adhere to it, and no adhesion is observed during retraction in this case. Such adhesion may occur if the tip was positioned over a monolayer defect and has been observed experimentally several times. The cantilever continues to deflect in a linear fashion (points 4 to 5) until the arbitrarily predefined deflection limit is reached, after which the piezo motion is momentarily ceased before being reversed.

During retraction the tip initially follows a similar linear deflection pathway in reverse (point 6). The tip then leaves the surface, after which there may be a small region where the tip is undeflected (point 7). This is immediately proceeded by the start of a nonlinear downward deflection of the cantilever as indicated by the negative sign of the deflection signal. The non-linearity during this region is attributed predominantly to the extension of the flexible PEG linking groups. After the piezo has retracted 36.5 nm in this case the cantilever reaches its maximum downward deflection of 2.6 nm (point 8) and abruptly returns to its undeflected state. As can be seen in sub-plot II the tip-surface separation immediately before the unbinding event occurs is slightly lower than the piezo displacement due to the downward deflection of the cantilever. After unbinding has occurred, the piezo continues to retract the cantilever but it experiences no more forces, since it is far away from the surface (point 9).

**Figure 6:**
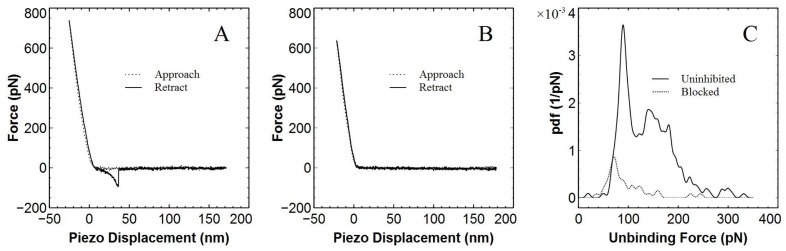
Measurement of interaction force between single biotin-avidin pairs with tips and substrates modified using the new protocol. **(A)** Example force-displacement curve showing specific biotin-avidin unbinding. The calculated most probable rupture force was 89±13 pN and the loading rate was 4091 ± 69 pN s^−1^. **(B)** Example force-displacement curve after blocking with free avidin. **(C)** Empirical probability density functions representing force distributions acquired without inhibition (solid line) and in the presence of free avidin (dotted line). Unbinding probabilities were 27% and 4% for uninhibited and blocked experiments respectively.

An example of a calibrated force-displacement curve depicting a specific unbinding event is shown in Figure 6A. The unbinding event in the retraction curve is clearly specific in nature due to the characteristic nonlinear extension immediately prior to tip detachment. Furthermore, non-specific adhesion which is usually manifested by a linear non-delayed extension just after the piezo displacement zero point [30,44] is negligible. Non-specific forces are typically problematic for force spectroscopy experiments as they can often be large and thus obscure specific force measurements. In addition, due to the large length of the linkers utilised for attachment, the unbinding event occurs several nanometres away from the surface, making it easily identifiable. For this representative example we calculated the unbinding force to be 87 ± 13 pN.

In order to determine the loading rate we fitted the worm-like-chain (WLC) model to the retraction curve (force versus tip-sample displacement) as described elsewhere [54]. The gradient at the point immediately prior to rupture is then equal to the spring constant of the flexible linker, *k*_PEG_. Thus the effective spring constant was determined by *k*_eff_ = (*k*_PEG_^−1^ + *k_c_*^−1^)^−1^, where *k_c_* is the cantilever spring constant and the loading rate found from *r* = *k*_eff_*v*, where *v* is the retraction speed. This was then repeated for each of the curves (~ 270) and an average loading rate determined to be 4091 ± 69 pN s^−1^. If a simple linear retraction profile had been assumed such that *r* = *k_c_v*, we would obtain *r* = 13200 pN s^−1^, which is a significant deviation from the correct value. The nonlinearity of the extension profile therefore clearly influences the loading rate in our case as reported elsewhere [55,56].

After blocking tip bound biotin by introducing free avidin into the buffer solution, the number of specific unbinding events recorded decreased significantly and many more force-displacement plots were of the form shown in Figure 6B. Empirical probability density functions were then constructed from the force distribution data as shown in Figure 6C. For each experiment, 1000 approach-retract cycles were conducted, out of which a certain number were found to contain specific unbinding events. Unbinding probabilities for this data set were 27% and 4% for uninhibited and blocked experiments respectively, with the area of each probability density function scaled to reflect this. The specificity tests were repeated for four different cantilevers each prepared with the new protocol, the results of which are summarised in Table 1. On average the unbinding probability changed from 18 ± 5% uninhibited to 7 ± 1% after blocking, which is a significant decrease.

**Figure 7:**
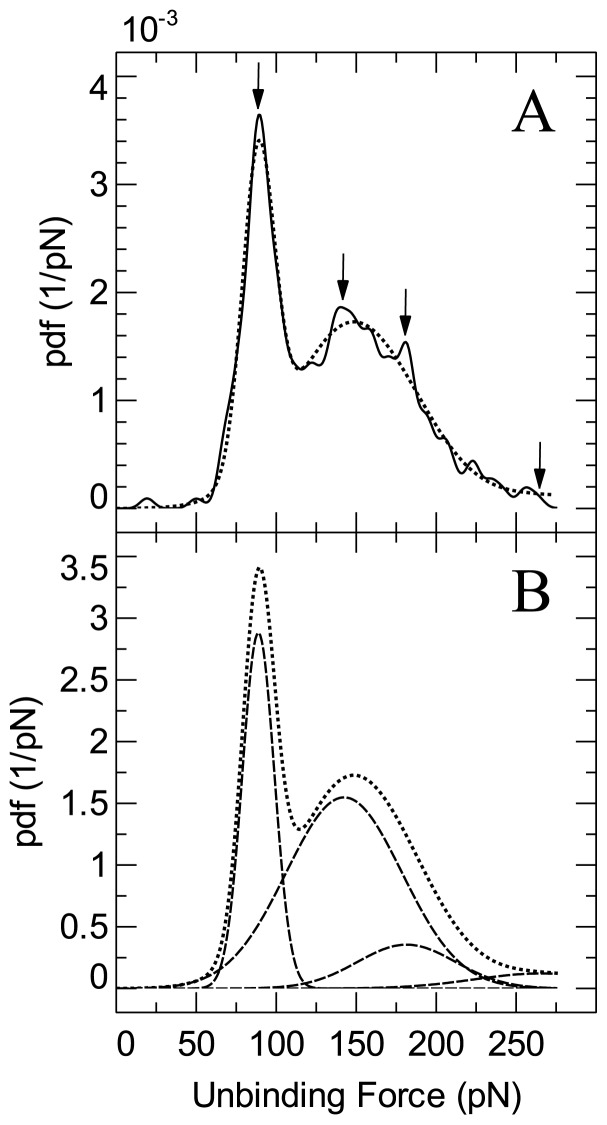
Peak analysis of probability density function corresponding to biotin-avidin data. **(A)** Raw data represented by solid line, model based on sum of four Gaussian distributions shown with a dotted line. The calculated peak values of 89 pN, 142 pN, 181 pN and 266 pN are indicated by arrows. **(B)** The four individual Gaussian components are shown (dashed lines) for clarity.

In order to more accurately determine the unbinding force of the biotin-avidin interaction for the particular loading rate studied, further data analysis was conducted on the uninhibited data set already presented in Figure 6C. As can be seen in Figure 7 the force distribution (solid line) is well fitted by the sum of four Gaussian peaks (dotted line). The individual Gaussian distributions had peaks of 89 pN, 142 pN, 181 pN and 266 pN (as indicated by arrows on the plot) with standard deviations of 10 pN, 36 pN, 31 pN and 42 pN. The second and third peaks are likely attributable to multiple simultaneous unbinding of ligand receptor pairs, which may occur if more than one biotin molecule is attached to the tip. The inclusion of the fourth peak is purely putative and has a large uncertainty. The first peak however seems to fit very well to the data attributable to single biotin-avidin unbinding. Based on the standard deviation of this Gaussian fit combined with the cantilever spring constant uncertainty, the peak force was determined to be 89 ± 13 pN. The reasonable distinguishability of the first peak as compared to the multivalent rupture force peaks in this case permit the use of the simple multicomponent analysis. A more thorough approach has recently been used however [56]. Unfortunately, complete elimination of the multiple simultaneous rupture events in the experiments proved difficult using the current protocol. Nevertheless, as long as the single event peak is distinguishable, this should not cause problems for most types of experiment. In practice, it may be very difficult to eliminate multiple unbinding unless an individual ligand is controllably isolated on the tip.

Since the most probable rupture force of a complex depends on the loading rate [4], direct comparison to other work in the literature is difficult unless either the unbinding force was measured at the same rate or a dynamic spectrum that encompasses the desired rate was obtained. In addition, we may only directly compare avidin-biotin and not streptavidin-biotin data, since although these complexes are very similar, several reports indicate that the local structure of the respective energy landscapes differ. Four accounts of the avidin-biotin interaction measured using the BFP [4] and AFM [5–7] recorded over comparable loading rates are presented in Table 2. In each case, we estimated the form of the best fit linear equation describing the rate dependence of the most probable rupture force F^*^ (*r*) for the loading rate regime most closely related to our work. From this we then determined the expected force at our specific loading rate, F^*^(*r* = 4091 pN s^−1^). We find that our value of 89 ± 13 pN is in agreement with Merkel *et al*. [4] (≈ 78 pN) and comparable to the work of De Paris [5] (≈ 56 pN). However the values estimated for Yuan [6] and Piramowicz [7] are approximately two and four times greater respectively in comparison to our value. A recent report [19] highlights the influence of temperature on the rupture force of the streptavidin-biotin bond. Performing a similar analysis of this data, we find for the same loading rate (*r* = 4091 pN s^−1^) that F^*^(*T* = 17 °C) ≈ 101 pN, F^*^(*T* = 24 °C) ≈ 80 pN and F^*^(*T* = 37 °C) ≈ 66 pN. Since in most studies reported to date the temperature has not been well controlled, this may explain some of the differences observed. Another report [27] suggests that the difference in rupture force observed by Yuan and Merkel is due to the complex being in the lowest and second lowest minima respectively before a force is applied. This also indicates that we may have probed the second lowest minima, although further work would be needed to test this hypothesis.

## 3. Experimental Section

### 3.1. Materials and Instrumentation

HS-(CH_2_)_11_-(O-CH_2_-CH_2_)_6_-NH_2_ (Alkyl-PEG-Amine, APA, purity *>* 95%) and HS-(CH_2_)_11_-(O- CH_2_-CH_2_)_3_- Biotin (biotin, purity *>* 95%) were purchased from Prochimia (Gdansk, Poland). HS-(CH_2_)_10_-CO-NH-(CH_2_)_2_(O-CH_2_-CH_2_)_7_-OH (Alkyl-PEG-Hydroxyl, APH, purity *>* 95%) and maleimidopropionyl-PEG-NHS (crosslinker, NPM, purity *>* 95%) were purchased from Polypure (Oslo, Norway). Avidin from egg white (purity ≥ 98%), 2-iminothiolane hydrochloride (Traut’s Reagent, purity ≥ 98%), phosphate buffered saline (PBS: 10mM phosphate, 138mM NaCl, 2.7mM KCl, pH 7.4), dimethyl sulfoxide (DMSO, anhydrous, purity ≥ 99.9%) and absolute ethanol (purity ≥ 99.9%) were purchased from Sigma-Aldrich. Immulon 2B 96-well plates were purchased from Fisher and 12 well plates were purchased from Corning. Epotek 377 epoxy was purchased from Promatech (Gloucestershire, UK) or John P. Kummer (Marlborough, UK). Silicon *<*100*>*, 500 *μ*m thick test grade wafers were purchased from Compart Technology (Peterborough, UK). Gold wire (99.99%) was obtained from Advent Research Materials (Oxford, UK) and muscovite mica from Agar Scientific (Essex, UK). Gold coated cantilevers from Olympus (Biolevers BL-RC150VB) and silicon cantilevers from MikroMasch (NSC36) were used for force measurements and imaging respectively. Tris buffered saline (TBS) was prepared with 50 mM Tris-HCl, 140 mM NaCl, pH 7.4. The enzyme substrate TMB (3,3′,5,5′-tetramethylbenzidine) was purchased from Thermo Scientific. ImmunoPure biotinylated horseradish peroxidase (biotin-HRP) was purchased from Thermo Scientific (product number 29139). Bovine Serum Albumin (BSA) was purchased from PAA Laboratories GmBH, Austria (product number K45-001).

### 3.2. Preparation of Template-Stripped Gold (TSG) Surfaces

Ultraflat gold substrates were prepared using a similar process to that described by Wagner *et al*. [50,57]. Briefly, freshly cleaved muscovite mica sheets were placed in our BOC Edwards Auto 306 evaporator and heated to 300 °C in situ from the rear through the sample holder, for approximately 6–12 h at a pressure less than 10^−6^ mbar. This was to outgas adsorbed water or any other volatile contaminants. Gold films were deposited first by evaporating 20 nm at a rate ~ 0.04 nms^−1^ followed by 180 nm at 0.1–0.2 nms^−1^ whilst maintaining 300 °C. Samples were annealed in the evaporator again at 300 °C typically for a further 6 h and allowed to cool naturally before removal from the chamber. The gold deposited mica sheets were cut into 15 × 25 mm^2^ pieces and 1 cm^2^ silicon stubs were glued to the gold side using Epo-tek 377 two-part epoxy. This ensures that the silicon stub is completely covered with gold. The samples were cured for approximately 48 h at 70 °C and the TSG samples were stored in their pre-stripped state for up to several months. Immediately prior to use the mica was stripped from the TSG samples by gently bending the excess gold-mica layer normal to the silicon stub. In the majority of cases this yielded a clean, flat gold substrate. For occasions when some mica remained on the surface, adhesive tape was used to remove it and the sample was then washed with 20 mL ethanol (4 times each with 5 mL in a pipette) and dried. In all cases samples were checked for conductivity prior to chemical modification. Typically the RMS (root-mean-square) roughness was measured to be less than 0.5 nm over 1 *μ*m^2^.

### 3.3. Monolayer Preparation

#### Substrate

A Freshly stripped TSG sample was loaded into the Substrate Incubation Device (SID) and 200 *μ*L of 1 mM APA in ethanol was added to the well. This was incubated for 1 h before being washed with 20 mL ethanol (4 times each with 5 mL in a pipette) and dried under a stream of nitrogen. Next, 200 *μ*L of 2 mM crosslinker in PBS (diluted from 100 mM stock in DMSO) was added to the well. After 1 h the well was washed with 20 mL PBS (4 times each with 5 mL in a pipette) to remove any unreacted crosslinker. A 200 *μ*L solution of 1 *μ*M avidin and 10 *μ*M Traut’s reagent was then added to the well and left for 1–2 h. The well was washed again with 20 mL PBS (4 times each with 5 mL in a pipette) and care taken to ensure that the proteins were not dehydrated.

#### Cantilevers

Cantilevers were stored in a cleanroom environment, washed with ethanol and dried under a stream of dry nitrogen immediately before use. A 100 *μ*L solution containing 0.1 mM APA and 0.9 mM APH (10% APA/APH ratio) in ethanol was added to one well of a 96-well plate, before carefully submerging a gold coated cantilever. After incubating the lever for 1 h it was carefully washed by dipping the cantilever in a petri dish containing ~5 mL of ethanol and gently agitating for several minutes, and allowed to dry by evaporation. Next, 100 *μ*L of 2 mM crosslinker in PBS (diluted from 100 mM stock in DMSO) was added to a fresh well and the lever again carefully submerged. Stock solutions are kept at −20 °C when not in use and can be kept for several months. After a further hour, the tip was carefully washed by dipping the cantilever in a petri dish containing ~5 mL of PBS and gently agitating for several minutes, and any excess buffer was drawn away via capillary action from the chip using cleanroom wipes. Finally, 100 *μ*L of 5 *μ*M biotin in PBS/ethanol (50:50) was added to a new well and the cantilever again submersed. After 1 h the cantilever was then carefully washed by dipping the cantilever in a petri dish containing ~5 mL of PBS and gently agitating for several minutes to remove any unbound molecules. Usually tips and substrates were prepared in parallel over no more than 3–4 h. Figure 1 shows a schematic that illustrates the steps described in detail above taken towards protein attachment.

### 3.4. Enzyme-linked Immunosorbent Assay (ELISA)

#### Standard adhesion assay

Wells of an Immulon-2 96-well plate were coated with 10 *μ*M avidin in PBS for 1 h at room temperature. Avidin was replaced with 10 *μ*gmL^−1^ BSA to produce a negative control. The wells were first blocked for 60 mins with 200 *μ*L of 5% BSA in TBS, then emptied and washed three times with adhesion buffer (TBS containing 0.1% BSA). The wells were then incubated with biotin-HRP at 1 *μ*gmL^−1^ for 1 h before washing 3 times with adhesion buffer. Next, 100 *μ*L TMB substrate was added to the wells and left for up to 60 min to allow oxidisation by HRP of the chromogen, TMB substrate, to a blue coloured product. To stop the reaction, 100 *μ*L of 2.5 M sulphuric acid was added to the wells, changing the colour to yellow, and the absorbance was read at 450 nm on a Molecular Devices Emax microplate reader.

#### Modified adhesion assay

Avidin bound glass slides were prepared by coating for 1 h with 50 *μ*L of 1 *μ*M avidin in PBS (drop-casting). Negative controls were prepared simply by coating glass slides with 50 *μ*L of TBS containing BSA at 10 *μ*gmL^−1^. Avidin bound TSG was prepared via the standard monolayer protocol, whereas unmodified TSG was used as a negative control. Freshly prepared biotin bound via the monolayer to TSG was washed with copious amounts of PBS, water and ethanol (~20 mL of each) before drying under a stream of dry nitrogen. These samples were then incubated in 1 *μ*M avidin for 1 h in the SID. To test for the presence of avidin coupled to TSG surfaces or glass slides coated with avidin, a new protocol based on the standard assay was devised. Samples were first transferred to the wells of a 12 well plate and blocked for 1 h (30 min each side) with 1 mL TBS containing 5% BSA and washed 3 times by successive dipping into beakers of adhesion buffer. Next, 1 mL of biotin-HRP (1 *μ*gmL^−1^) was added and incubated for 1 h followed by washing 3 times with TBS containing 0.1% BSA. Samples were then placed into fresh wells and 1 mL of TMB added to each well. These were then incubated for approximately 1 h. Finally 100 *μ*L of the reacted TMB solution was added to a clean 96-well plate and 100 *μ*L of 2.5 M sulphuric acid added to stop the reaction. Again the absorbance was read at 450 nm.

### 3.5. Atomic Force Microscopy

A PicoPlus AFM with a PicoSPM II controller from Molecular Imaging was used for all force spectroscopy and imaging work. Cantilever spring constants were estimated using the Sader Method [38]. Briefly, thermal noise spectra were acquired using an MFP-3D AFM from Asylum Research and the equation of a simple harmonic oscillator fitted to extract the fundamental resonance frequency and the quality factor. Length and width dimensions of the rectangular cantilevers were measured using an optical microscope. The analysis typically yielded spring constant values about 0.03 Nm^−1^, consistent with nominal values quoted by the manufacturer. Calibration of gold coated cantilevers was always performed before any chemical modification.

All force spectroscopy measurements were conducted in a fluid cell in PBS. Modified tips were repeatedly approached towards and retracted away from the modified substrate in a cyclic manner whilst simultaneously monitoring the deflection signal using a four quadrant photodiode. Amplitudes were typically 200nm with a frequency of 1 Hz. Approach-retract cycles were performed 1000 times with each tip for each experiment across five different areas of the substrate. Typically an uninhibited experiment would be conducted first, followed by a blocking experiment in which free avidin was introduced into the fluid cell. Force-distance cycles were analysed using scripts that we developed for Matlab version R2007a (MathWorks Inc., Natick, MA, USA). Briefly, an algorithm first identifies any data points which deviate significantly from the rest of the data by comparing the gradient between successive points to the standard deviation of the gradient of all points. The user then chooses an event if it is preceded by a nonlinear force-displacement retraction profile that is unimpeded by any other interaction. If no such event meets this criteria the data set is discarded. Empirical Probability density functions [39,54] were then constructed from the distribution of unbinding forces. This representation is advantageous over simple histograms since each data point is weighted by their accuracy (essentially governed by the thermal noise of the cantilever fluctuations) and therefore yields better resolution.

For AFM imaging of substrates, TSG samples with covalently bound monolayers were first washed with 20 mL ethanol (4 times each with 5 mL in a pipette), or 20 mL water (4 times each with 5 mL in a pipette) for the case of protein bound surfaces, and dried under a stream of dry nitrogen. They were then imaged using AC mode in air with the silicon cantilevers from Mikromasch (nominal spring constant 1.75 Nm^−1^).

### 3.6. Quartz Crystal Microbalance Assay

Measurements were made using a Q-sense E4 instrument (Q-Sense, Sweden) with standard gold coated quartz crystal sensors (QSX 301). The fundamental resonant frequency (5 MHz) and several harmonics (*n* = 3; 5; 7) were recorded.

After loading the sensor, an ethanol/water (50:50) solution was first drawn into the chamber and left for ~ 12 h so as to achieve a stable baseline. We found that the sensitivity of the QCM to the detection of bonding of APA to gold was less sensitive in ethanol than with the ethanol/water mixture, and the APA appears soluble in both. Next 300 *μ*L of 1 mM APA in ethanol/water (50:50) was drawn into the chamber and left for ~ 60 min. The chamber was again flushed with ethanol/water to wash away all unreacted material and left for several minutes until a stable baseline was achieved. The chamber solvent was then changed to PBS, which resulted in a large shift in absolute frequency as expected. After a stable baseline was obtained, 300 *μ*L of 2 mM NPM was drawn into the chamber and allowed to react with the surface bound amine groups for ~ 30 min. After this the chamber was again washed with PBS before finally adding a 300 *μ*L solution of 1 *μ*M avidin (previously reacted with a 10-fold excess of Traut’s reagent for 10 min) and incubating for ~ 1.5 h after which a final washing step was performed using PBS.

## 4. Conclusions

In this paper we have reported on the development of a protocol for the attachment of biomolecules to gold coated tips and ultraflat gold substrates for use in single molecule force spectroscopy experiments. The three stage process utilises mixed self-assembled monolayers containing both active and passive components, to which the former are coupled to sulfhydryl groups on target proteins via a heterobifunctional crosslinker containing both *N*-hydroxysuccinimide (NHS) and maleimide functionality. Poly(ethylene) glycol (PEG) groups were used in a passivation role to both reduce non-specific interaction forces and provide essential mobility for coupled ligand-receptor pairs. As demonstrated previously [44], by varying the ratio of the different alkanethiol components, the surface density of bound biomolecules is well controlled, which is essential for single molecule measurements. Furthermore, site-directed coupling is possible provided a suitably positioned cysteine residue is accessible on the target molecule. However, almost any protein is amenable for attachment with the protocol by simple pre-treatment with Traut’s Reagent.

The new attachment protocol was tested by probing the interaction force between the model ligand-receptor pair, biotin-avidin. We measured a most probable rupture force between biotin and avidin of 89 ± 13 pN at a loading rate of 4091 ± 69 pN s^−1^, comparable to previously reported values measured at similar loading rates [4,5]. Specificity tests showed that on average the unbinding probability changed from 18 ± 5% uninhibited to 7 ± 1% after blocking, indicating the measurement of a specific biological interaction.

## Figures and Tables

**Table 1:** Results of specificity tests for the biotin-avidin interaction for four different cantilevers each prepared using the new protocol in the absence (uninhibited) or presence (blocked) of free avidin.

Tip	Experiment	Recognition,%
A	uninhibited	27
B	uninhibited	25
C	uninhibited	6
D	uninhibited	15
A	blocked	4
B	blocked	8
C	blocked	10
D	blocked	6

**Table 2:** Comparison of most probable rupture forces obtained for the biotin-avidin complex.

Reference	Rate regime ( pN s^−1^)	F*(*r*) ( pN)	F^*^(*r =* 4091) ( pN)
Merkel *et al*. [4]	10^2^ to 10^4^	14 ln(*r*) – 38	≈ 78
De Paris *et al*. [5]	10^3^ to 10^4^	15.5 ln(*r*) – 71.4	≈ 56
Yuan *et al*. [6]	1000 to 5000	20.6 ln(*r*) – 12.4	≈ 157
Piramowicz *et al*. [7]	1700 to 9600	168.6 ln(*r*) – 1047.6	≈ 355
Current work	4091 ± 69	-	89 ± 13 pN
